# Providing Mobile Patient Access to Their Electronic Secondary Care Patient Record in Adults With Cystic Fibrosis: Results of a Prospective, Parallel, Randomized Open-Pilot Quantitative Study

**DOI:** 10.2196/69747

**Published:** 2025-12-25

**Authors:** Helen K Chadwick, Akhil Sawant, Helen White, Lindsey Gillgrass, Giulia Spoletini, Ian J Clifton, Christine Etherington, Daniel G Peckham

**Affiliations:** 1 Respiratory Medicine Leeds Institute of Medical Research at St James's University of Leeds Leeds, West Yorkshire United Kingdom; 2 Adult Cystic Fibrosis Unit St James's University Hospital Leeds Teaching Hospitals NHS Trust Leeds, West Yorkshire United Kingdom; 3 School of Clinical & Applied Science Leeds Beckett University Leeds, West Yorkshire United Kingdom

**Keywords:** Patient Access, anxiety, quality of life, questionnaires, electronic health care records

## Abstract

**Background:**

The Leeds regional adult and pediatric cystic fibrosis (CF) services introduced a modified primary care electronic health care record (EHR) in 2007. This resulted in a dramatic improvement in efficiency while providing the benefits of primary care developments, including full Patient Access to their records.

**Objective:**

This study aims to evaluate the feasibility, benefits, usability, and acceptability to patients of providing secure access to linked secondary care in CF.

**Methods:**

A prospective, parallel, randomized, open, pilot study with an intervention (EHR access; I) and a control group (no EHR access; C). People with CF were recruited on a consecutive basis, from outpatient clinics or as inpatients on the regional Leeds adult CF unit. At baseline and 6 months, paper-based self-report questionnaires were completed by participants to assess having access to EHR on psychological impact, patient satisfaction, quality of life (QoL), patient and physician relationships, and patterns and rates of adherence to treatment. Perceptions and engagement with Patient Access and computer literacy were also assessed. Once completed, participants were randomized into either the intervention or control group (1:1), with those in the intervention group given instructions about how to gain access and the functions of Patient Access by the research team.

**Results:**

A total of 91 people with CF completed the 6-month study (intervention n=45; median age 27.5, IQR 12.0 years; 22 male participants; control group n=46; median age 27.0, IQR 15.0 years; 29 male participants). Median number of logins was 9 (range 1-205). There was no effect of Patient Access on levels of anxiety (Generalized Anxiety Disorder-7; I=3.0, C=5.0), all symptom QoL scales and seven QoL domains (Cystic Fibrosis Questionnaire-Revised; respiratory I=63.89, C=55.56; weight I=100.00, C=66.67; digestion I=88.89, C=88.89; physical I=60.42, C=50.00; vitality I=54.17, C=41.67; emotional I=86.67, C=66.67; role I=75.0, C=75.0; body image I=77.78, C=66.67; eating disturbances I=88.89, C=100.0; treatment burden I=55.56, C=55.56), levels of depression (Patient Health Questionnaire-9; I=3.0, C=7.0), confidence in managing health care (Patient Activation Measure-13; I=66.67, C=60.63), level of trust in health care professionals (patient and provider perceived efficacy in patient-physician interaction; I=49.0, C=47.0), and computer literacy. Patient Access scored 86% for satisfaction, 82% for ease of use, and 80% for usefulness (Perceived Health Web Site Usability Questionnaire). Of those who had EHR access, 41 of 42 (98%) participants agreed that access to EHR should continue.

**Conclusions:**

This pilot study suggests that providing access to EHR in adults with CF does not appear to have a negative effect (increase levels of anxiety or decrease QoL), and uptake by patients has been very positive. Prospective studies are needed to investigate the long-term effect on objective health outcomes and how we can improve the functionality of such apps from the patient perspective.

**Trial Registration:**

ClinicalTrials.gov NCT06122025; https://clinicaltrials.gov/study/NCT06122025

## Introduction

Over the past decade, a revolution in technology has fundamentally changed the way people live and how they access information. This new digital age has resulted in an exponential growth in social media and web-based access to services such as banking and shopping. The COVID-19 pandemic resulted in access to digital health solutions being implemented and adopted at unprecedented rates [1]. While digital technology can transform the way patients engage in health care, misinformation, misinterpretation, and information overload have the potential to negatively impact physical and mental health [1-4].

The National Health Service and other national health care services have recognized the importance of a more joined-up working with an integrated approach to health care services. This has the potential to improve safety and efficiency, clinical standards, and disease prevention, and empowers users to take control of their own health [3,5-7].

The vision for primary care was that all citizens should have digital access to their general practitioner records by 2015 [8]. This vision has been successful, and by 2017, 95% of general practitioner practices were able to offer digital access to detailed primary care records, including test results. Since 2020, all health care users are now able to access the full record, including note annotations, largely due to the COVID-19 pandemic and the significant improvement in remote health services [1,9]. This new transparency in record sharing has met some resistance from health care professionals due to concerns that accessing unfiltered medical information may lead to increased workload, patient anxiety, and potential litigation [3,10-12]. Equally, some patients have expressed anxiety over the potential for breaches in security and confidentiality [4].

In contrast to primary care, full digital access in secondary care is lagging [13], limited by the accessibility of platforms and the fragmentation and lack of digitized records.

In Leeds, the regional adult and pediatric cystic fibrosis (CF) services introduced a modified primary care electronic health care record (EHR) in 2007 and went paperless. This resulted in a dramatic improvement in efficiency while providing the benefits of primary care developments, including full Patient Access to their records [14]. However, the multidisciplinary team had concerns that providing patients with full access to their records might increase anxiety and affect the way the team recorded information. Therefore, prior to opening the portal to all patients, we performed a pilot study to evaluate the feasibility, benefits, and acceptability to patients of providing secure access to linked secondary care in CF. We also wanted to explore technological usability and the impact of the shared records on communication and patient satisfaction. It was hypothesized that providing Patient Access would have an effect on levels of anxiety and quality of life (QoL) scores in the intervention group.

## Methods

### Design

A prospective, parallel, randomized, open, pilot study was conducted between April 2018 and May 2019 (ClinicalTrials.gov Identifier: NCT06122025).

People with CF were recruited on a consecutive basis, either from routine CF outpatient clinics (Seacroft Hospital, Leeds, UK) or as an inpatient on the regional Leeds adult CF unit (St James’s University Hospital, Leeds, UK). Eligible people were identified using the Leeds adult CF unit EHRs [14]. Inclusion criteria consisted of diagnosis of CF (confirmed by the presence of a CF phenotype with either two CF-causing mutations or a single mutation with two positive sweat chloride [>60 mmol/L]), aged 17 years or older, able to give written informed consent, and presence of an EHR system at the regional Leeds adult CF unit. Patients were excluded if they were taking part in a clinical trial.

Once consented, individuals were allocated to either the intervention or control group (1:1 ratio) by the research team using a randomized sampling technique. Random sequenced numbers were generated with the smallest value of 1 and the largest of 100, and even numbers were assigned to the intervention.

### Measures

#### Overview

A baseline paper-based self-report questionnaire was used to collect demographic data, including age and gender.

Participants completed paper-based self-report questionnaires at week 0, and again at week 26±1, to evaluate the psychological impact, and effect on patient satisfaction, QoL, patient and physician relationships, and patterns and rates of adherence to treatment of having access to EHR in secondary care. Perceptions and engagement with Patient Access, and computer literacy were also assessed.

#### Primary Outcome Measures

##### Level of anxiety

The Generalized Anxiety Disorder-7 (GAD-7) is a validated questionnaire, further validated for use in CF, to assess the level of anxiety through seven items covering the preceding 2 weeks [15,16].

##### QoL

The Cystic Fibrosis Questionnaire-Revised (CFQ-R) was used to assess QoL [17,18].

#### Secondary Outcome Measures

##### Severity of Depression

The Patient Health Questionnaire-9 (PHQ-9) is a validated questionnaire to measure the severity of depression [19], further validated for use in CF [16].

##### Knowledge, Skills, and Confidence in Managing Health

The Patient Activation Measure-13 (PAM-13) is a validated questionnaire to assess the knowledge, skills, and confidence a person with CF has in managing their health care [20].

##### Level of Trust and Interaction With Health Care Professionals

The patient and provider perceived efficacy in patient-physician interaction (PEPPI) is a validated questionnaire to assess the participants’ level of trust and interaction with their health care professionals [21].

##### Computer Literacy

This was assessed using a questionnaire modified from the “My diabetes, my way” survey to assess types, frequency, and experience of computer or internet use.

##### Perception of and Intention to Engage With Patient Access

A questionnaire was devised to assess patients’ thoughts about having access to their medical records in terms of reasons for accessing the records, expected effect of having access to them, and barriers to acceptance. This questionnaire was based on a modified OpenNotes presurvey [22] and the Physician and Patient Attitudes toward Technology in Medicine survey [23,24].

##### Perception of and Engagement With Patient Access (Intervention Group Only)

This questionnaire contained items from the baseline questionnaire modified to reflect having had access to their records for 6 months.

##### Satisfaction, Ease of Use, and Usefulness of Patient Access

The Perceived Health Web Site Usability Questionnaire (PHWSUQ; intervention group only) [25] was used to assess satisfaction, ease of use, and usefulness of the intervention.

##### Use Patterns

For the intervention group, data relating to the frequency of logins to their records was collected from the system audit trail.

A detailed description of the questionnaires used is available in Multimedia Appendix 1.

### Procedures

Eligible adults with CF were approached face-to-face at either routine CF outpatient clinics (Seacroft Hospital, Leeds, UK) or as an inpatient on the regional Leeds adult CF unit (St James’s University Hospital, Leeds, UK). If they expressed interest, they were provided with a participant information sheet by a member of the research team (who was also part of their direct care team), the study was explained to them, and they had the opportunity to ask questions and have them answered satisfactorily. Upon enrollment, participants completed the paper consent form and baseline paper-based self-report questionnaires in the following order: GAD-7, PHQ-9, PAM-13, CFQ-R, PEPPI, computer literacy, and “perceptions of and intention to engage with Patient Access.” Participants were then randomized into either the intervention or control group. Those in the intervention group were instructed in how to gain access, and the functions of Patient Access were explained to them by a member of the research team (who was also part of their direct care team); access to current problems and medication, test requests, letters, consultations, allergies, and immunizations. Information about Patient Access security and privacy was also explained. No prompts were used in the study; those in the intervention used Patient Access at their discretion during the 6-month study period. Help and support were provided only if the participant requested them. After 26 (+/–1) weeks, participants completed the follow-up paper-based questionnaires (GAD-7, PHQ-9, PAM-13, CFQ-R, PEPPI, and computer literacy). Those in the intervention group also completed the end of intervention “Perception of Patient Access and their engagement” questionnaire and PHWSUQ on paper. Following successful completion of the questionnaires, participants in the control group were granted access to their EHR if they expressed interest.

### Ethical Considerations

The study was approved by London—Camberwell St Giles Research Ethics Service (17/LO/19; 26/11/2017). Informed consent was obtained prior to any study-specific assessments. A participant information sheet and consent form were provided to potential participants, the content of the document was fully explained to them, and they were given enough time to consider the information. Participants had the opportunity to ask any questions about the study, and answers were provided to their satisfaction. A signed and dated copy of the informed consent form was provided to participants. All participants were provided with a unique study identifier after informed consent so that study data were anonymous. No compensation was provided for study participation.

### Statistical Analysis

An a priori sample size calculation was performed using the following formula, assuming a 2-sided 5% significance level, a standardized effect size of 0.6, 80% power, and a dropout rate of 10%. The calculation indicated that 100 participants would be required, allocated to group on a 1 intervention:1 control, resulting in 50 individuals being recruited to the intervention (given mobile access to their own secondary care EHR) and 50 people to the control group (no access to their secondary care EHR).

Analysis was conducted separately for participants with data at both baseline and follow-up. Descriptive statistics were produced for computer literacy. Data were expressed as a number or median (IQR) as appropriate. For proportions, a Fisher exact test was used, and for measures, a Mann-Whitney *U* test was used for between-subjects. No correction for multiplicity was applied. *P*<.05 was used as a guide to significance, and all computations used SPSS (v26.0 or higher; IBM Corp).

## Results

### Participants’ Characteristics at Baseline

**Figure 1:**
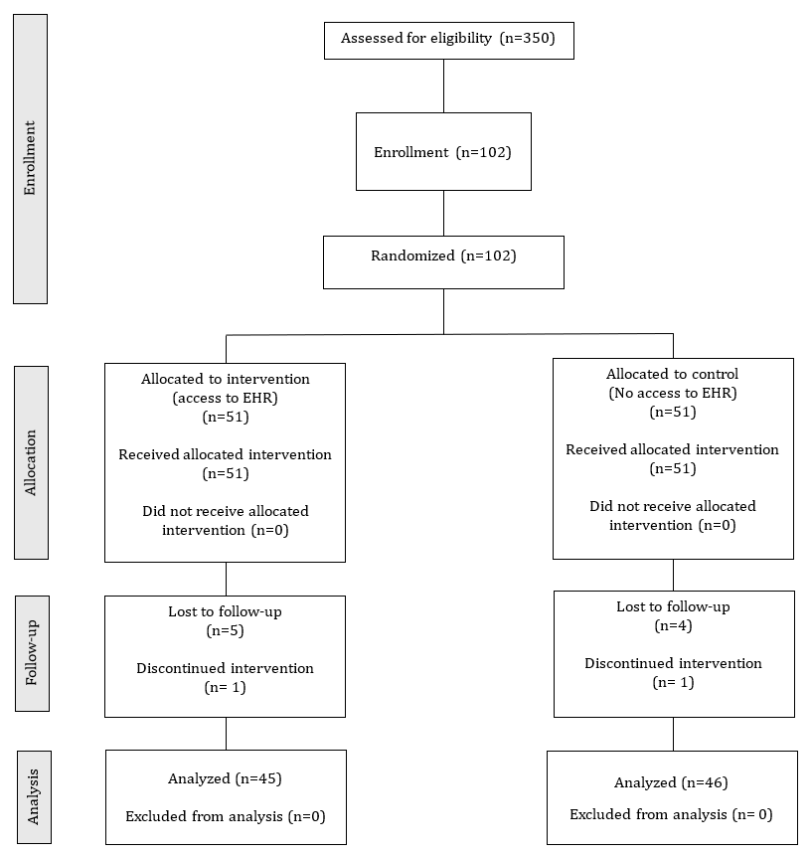
CONSORT flow diagram for patient enrollment, allocation, follow-up, and analysis. CONSORT: Consolidated Standards of Reporting Trials; EHR: electronic health care record.

A total of 350 people met the inclusion criteria. In total, 102 patients were recruited, 51 in each group (Figure 1). An extra individual was recruited to each group to account for two people who dropped out shortly after consent and randomization, respectively. Baseline characteristics are shown in Table 1.

**Table 1:** Questionnaire scores for levels of anxiety (GAD-7^a^), QoL^b^ (CFQ-R^c^), depression (PHQ-9^d^), knowledge, skills, and confidence in managing health (PAM-13^e^), and level of trust and interaction with health care professionals (PEPPI^f^), at baseline and follow-up (6 months) for the intervention and control groups.

	Baseline, mean (SD)				Follow-up, mean (SD)		
	Intervention group	Control group	*P* value	Intervention group		Control group	*P* value
Anxiety level (GAD-7)	4.0 (5.0)	3.0 (7.0)	≥.99	3.0 (5.0)		5.0 (5.0)	≥.99
QoL physical functioning (CFQ-R)	62.50 (51.04)	45.83 (62.50)	.37	60.42 (65.63)		50.00 (62.50)	.48
QoL vitality (CFQ-R)	50.0 (35.42)	41.67 (25.0)	.76	54.17 (41.67)		41.67 (41.67)	.35
QoL emotional functioning (CFQ-R)	73.33 (26.67)	66.67 (93.33)	.17	86.67 (38.33)		66.67 (43.33)	.22
QoL social functioning (CFQ-R)	72.22 (22.22)	61.11 (33.33)	<.001	66.67 (27.78)		61.11 (36.11)	.65
QoL role functioning (CFQ-R)	75.00 (33.33)	66.67 (33.33)	.15	75.00 (41.67)		75.00 (45.83)	.38
QoL body image (CFQ-R)	66.67 (55.56)	66.67 (44.44)	.29	77.78 (33.33)		66.67 (44.44)	.25
QoL eating disturbances (CFQ-R)	100 (22.22)	88.89 (22.22)	.30	88.89 (22.22)		100.0 (33.33)	.53
QoL treatment burden (CFQ-R)	55.56 (25.00)	55.56 (33.33)	.81	55.56 (33.33)		55.56 (38.89)	.33
QoL respiratory symptoms (CFQ-R)	55.56 (29.17)	61.11 (27.78)	.74	63.89 (33.33)		55.56 (38.39)	.90
QoL health perceptions (CFQ-R)	61.11 (33.33)	44.44 (44.44)	.04	55.56 (44.44)		55.56 (38.89)	.79
QoL weight (CFQ-R)	83.33 (66.67)	66.67 (66.67)	.14	100.0 (58.33)		66.67 (66.67)	.20
QoL digestion (CFQ-R)	88.89 (22.22)	88.89 (33.33)	.68	88.89 (3.33)		88.89 (33.33)	.34
Depression level (PHQ-9)	4.0 (6.0)	6.0 (8.0)	.17	3.0 (7.8)		7.0 (10.5)	.05
PAM-13	65.47 (17.53)	65.47 (19.20)	.24	66.65 (17.11)		60.63 (19.36)	.05
PEPPI	46.00 (10.00)	44.00 (10.00)	.26	49.00 (9.0)		47.00 (8.0)	.38

^a^GAD-7: Generalized Anxiety Disorder-7.

^b^QoL: quality of life.

^c^CFQ-R: Cystic Fibrosis Questionnaire-Revised.

^d^PHQ-9: Patient Health Questionnaire-9.

^e^PAM-13: Patient Activation Measure-13.

^f^PEPPI: patient and provider perceived efficacy in patient-physician interaction.

Groups were similar for age (intervention: 27.5, IQR 14.0 years vs control 27, IQR 15.0 years; *P*=.96) and gender distribution (intervention 27 male participants, control 32 male participants; *P*=.32).

At baseline, there was no difference between groups for levels of anxiety (GAD-7), the three-symptom scales (respiratory, weight, and digestion), and seven of the nine QoL (CFQ-R) domains (physical, vitality, emotional, role, body image, eating disturbances, and treatment burden), levels of depression (PHQ-9), knowledge, skills, and confidence a person with CF has in managing their health care (PAM-13), or their level of trust in and interactions with their health care professionals (PEPPI; Table 1). Those in the intervention group had higher levels of health perceptions and social functioning (*P*=.04 and *P*<.001, respectively). One participant in the intervention group did not complete the baseline computer literacy questionnaire. Computer literacy was similar across groups (Table 2).

**Table 2:** Assessment of computer skills (literacy) for the intervention and control group at baseline and follow-up (6 months).

		Baseline, n (%)		Follow-up, n (%)	
		Intervention	Control	Intervention	Control
**How would you describe your computer skills?^a^**					
	Poor	0 (0)	2 (4)	0 (0)	3 (6)
	Moderate	2 (4)	2 (4)	3 (7)	3 (6)
	Average	14 (28)	11 (22)	10 (22)	9 (20)
	Good	13 (26)	13 (25)	15 (33)	15 (33)
	Very good	21 (42)	23 (45)	17 (38)	16 (35)
**Which of the following devices do you own?^a^**					
	Desktop computer	13 (26)	10 (20)	14 (31)	13 (28)
	Laptop computer	23 (46)	23 (45)	33 (73)	30 (65)
	“Internet-ready” smartphone	46 (92)	49 (96)	40 (89)	43 (93)
	Computer tablet (eg, iPad)	35 (70)	32 (63)	28 (62)	25 (54)
**How often do you use these devices?^a^**					
	Everyday	48 (96)	47 (92)	44 (98)	45 (98)
	Several times a week	0 (0)	3 (6)	1 (2)	0 (0)
	Several times a month	2 (4)	0 (0)	0 (0)	1 (2)
	Less than monthly	0 (0)	1 (2)	0 (0)	0 (0)

^a^One intervention participant did not complete the question at baseline.

At baseline, 48 of 50 (96%) participants assigned to the intervention and 46 of 50 (92%) controls agreed that Patient Access was a good idea (Table 3). In terms of access, 45 of 50 (90%) patients in the intervention and 42 of 50 (84%) controls thought that patients have a right to see all consultations (rather than health care professionals only sharing what they think is appropriate) and the majority of each group thought that having access would improve the patient-health care relationship (n=47 intervention, n=43 control). Reasons for wanting access to their records were because they were curious (n=29 intervention, n=33 control), they wanted to see test results (n=40 intervention, n=36 control), they wanted to know about their health (n=44 intervention, n=40 control), and they had a right to see what was in their record (n=23 intervention, n=30 control). Over half of the individuals gave no particular reason for not wanting to access their record (n=28 intervention, n=27 control), whereas the most popular specific reasons were worrying about the privacy of information (n=6 intervention, n=8 control) and because they were afraid about finding something negative about their health which they were not aware about (n=6 intervention, n=8 control).

**Table 3:** Perceptions of and intention to engage with Patient Access (based on a modified OpenNotes presurvey and the Physician and Patient Attitudes toward Technology in Medicine survey) for the intervention and control group at baseline; reasons for accessing the records, expected effect of having access, and barriers to acceptance^a^.

		Intervention, n (%)	Control, n (%)	*P* value	
**In general, making EHRs^b^ available to people with CF^c^ (ie, having Patient Access) is a good idea?**					.74
	Disagree	0 (0)	0 (0)		
	Somewhat disagree	0 (0)	0 (0)		
	Somewhat agree	2 (4)	3 (6)		
	Agree	48 (96)	46 (92)		
	Don’t know	0 (0)	1 (2)		
**How much access should patients have to consultations?**					.67
	A patient has a right to see them all	45 (90)	42 (84)		
	Health care professionals should only share what they think is appropriate	5 (10)	8 (16)		
**Why might you like to be able to read your EHRs (tick or cross all that apply)?**					
	I am curious	29 (58)	33 (66)		
	I have the right to see what’s in my medical record	23 (46)	30 (60)		
	I want to see the diagnosis or test results	40 (80)	36 (72)		
	I want to check the notes for accuracy	11 (22)	10 (20)		
	I want to know about my health	44 (88)	40 (80)		
	Other	9 (18)	1 (2)		
**Why might you NOT like to be able to access your EHRs (tick/cross all that apply)?**					
	I don’t think it would be useful	0 (0)	0 (0)		
	I do not use the internet very much	0 (0)	0 (0)		
	I worry about the privacy of information	6 (12)	8 (16)		
	I do not need to see test results	0 (0)	1 (2)		
	I do not want to think about my health any more than I have to	1 (2)	8 (16)		
	I am afraid I will find out something bad about my health that I didn’t know	6 (12)	8 (16)		
	I do not need to see what health care professionals wrote about my visit	2 (4)	0 (0)		
	No particular reason	28 (56)	27 (54)		
	Other	2 (4)	4 (8)		
**Do you think you might show or discuss your EHRs with other people?**					.13
	Yes	38 (76)	27 (54)		
	No	4 (8)	10 (20)		
	Don’t know	8 (16)	13 (26)		
**With whom do you think you might share your EHRs (tick/cross all that apply)?**					
	Partner	40 (80)	28 (56)		
	Family friend or relative	36 (72)	32 (64)		
	Friend	11 (22)	8 (16)		
	Doctor (outside of cystic fibrosis)	20 (40)	19 (38)		
	Nurse or Health care professional (outside of cystic fibrosis)	16 (32)	11 (22)		
	Someone else	5 (10)	2 (4)		
**Having access to my EHR would improve the patient-health care professional relationship?**					.54
	Agree	47 (94)	43 (86)		
	Disagree	3 (6)	6 (12)		
	Don’t know	0 (0)	1 (2)		
**I would better understand my health and medical conditions**					.48
	Disagree	1 (2)	1 (2)		
	Somewhat disagree	2 (4)	3 (6)		
	Somewhat agree	15 (30)	14 (28)		
	Agree	32 (64)	28 (56)		
	Don’t know	0 (0)	4 (8)		
**I would better remember the plan for my care**					.69
	Disagree	1 (2)	2 (4)		
	Somewhat disagree	0 (0)	2 (4)		
	Somewhat agree	10 (20)	11 (22)		
	Agree	38 (76)	33 (66)		
	Don’t know	1 (2)	2 (4)		
**I would feel more in control of my health care**					.28
	Disagree	1 (2)	4 (8)		
	Somewhat disagree	1 (2)	4 (8)		
	Somewhat agree	11 (22)	9 (18)		
	Agree	37 (74)	31 (62)		
	Don’t know	0 (0)	2 (4)		
**I would worry more**					.70
	Disagree	25 (50)	22 (44)		
	Somewhat disagree	14 (28)	13 (26)		
	Somewhat agree	5 (10)	8 (16)		
	Agree	0 (0)	2 (4)		
	Don’t know	6 (12)	5 (10)		
**I would be concerned about my privacy**					.60
	Disagree	30 (60)	25 (50)		
	Somewhat disagree	10 (20)	12 (24)		
	Somewhat agree	10 (20)	10 (20)		
	Agree	0 (0)	1 (2)		
	Don’t know	0 (0)	2 (4)		
**I would be concerned about security of my record**					.53
	Disagree	28 (56)	22 (44)		
	Somewhat disagree	9 (18)	12 (24)		
	Somewhat agree	13 (26)	13 (26)		
	Agree	0 (0)	1 (2)		
	Don’t know	0 (0)	2 (4)		
**The information would be more confusing than helpful**					.75
	Disagree	25 (50)	21 (42)		
	Somewhat disagree	16 (32)	15 (30)		
	Somewhat agree	5 (10)	5 (10)		
	Agree	1 (2)	1 (2)		
	Don’t know	3 (6)	8 (16)		
**It could make my doctor’s job more difficult**					.60
	Disagree	17 (34)	21 (42)		
	Somewhat disagree	18 (36)	14 (28)		
	Somewhat agree	9 (18)	5 (10)		
	Agree	0 (0)	1 (2)		
	Don’t know	6 (12)	9 (18)		
**Learning how to use and access to my EHR will be easy for me**					.99
	Strongly disagree	2 (4)	2 (4)		
	Disagree	1 (2)	1 (2)		
	Neither agree nor disagree	4 (8)	5 (10)		
	Agree	19 (38)	18 (36)		
	Strongly agree	24 (48)	23 (46)		
**I have the resources necessary to use and have access to my EHR (eg, the internet)**					.16
	Strongly disagree	3 (6)	1 (2)		
	Disagree	0 (0)	0 (0)		
	Neither agree nor disagree	0 (0)	4 (8)		
	Agree	10 (20)	8 (16)		
	Strongly agree	37 (74)	36 (72)		
**I plan to use and access to my EHR in the next 6 months**					.02
	Strongly disagree	2 (4)	2 (4)		
	Disagree	0 (0)	2 (4)		
	Neither agree nor disagree	1 (2)	8 (16)		
	Agree	17 (34)	7 (14)		
	Strongly agree	30 (60)	30 (60)		

^a^One intervention participant and one control participant did not complete the question at baseline.

^b^EHR: electronic health care record.

^c^CF: cystic fibrosis.

Over 50% (38/50 intervention; 27/50 control) of patients in each group thought that they might show or discuss their records with other people, such as their partner (n=40/50 intervention, n=28/50 control) or family friend or relative (n=36/50 intervention, n=32/50 control).

Prior to being randomized, the majority agreed (reporting either somewhat agreed or agreed) that they plan to use and access their records for the intervention period (n=47 intervention, n=37 control), that having access would help them understand their health and medical conditions (n=47 intervention, n=42 control), they would remember their care plan better (n=48 intervention, n=44 control), feel more control of their health care (n=48 intervention, n=40 control), and that learning how to use Patient Access would be easy for them (n=43 intervention, n=41 control) because they had the resources (n=47 intervention, n=44 control). The majority also disagreed (reporting either somewhat disagreed or disagreed) that as a result of having access, they would worry more (n=39 intervention, n=35 control), be concerned about privacy (n=40 intervention, n=37 control), or security (n=37 intervention, n=34 control), information would be more confusing than helpful (n=41 intervention, n=36 control), and it would make the doctor’s job more difficult (n=35 intervention, n=35 control).

### Follow-Up

In total, 91 patients completed the 6-month study. No differences in age or gender were observed across the two groups, considering only those who had completed the study (intervention n=45; median age 27.5, IQR 12.0 years; 22 male participants; control group n=46, median age 27.0, IQR 15.0 years; 29 male participants; *P*=.17).

There was no evidence of an effect of Patient Access on levels of anxiety (GAD-7), the three symptom QoL scales (respiratory *P*=.90; weight *P*=.20; and digestion *P*=.39), seven of the nine QoL (CFQ-R) domains (physical *P*=.48; vitality *P*=.35; emotional *P*=.22; role *P*=.38; body image *P*=.25; eating disturbances *P*=.53; and treatment burden *P*=.33), levels of depression (PHQ-9), confidence in managing health care (PAM-13), their level of trust in health care professionals (PEPPI), or computer literacy (Tables 1 and 2). The QoL domain “social functioning” decreased by six points in the intervention but remained stable in the control group. The health perceptions domain (CFQ-R) decreased in the intervention by six points but increased in the control group by nine points at follow-up.

In the intervention group, 41 of the 42 (98%) participants agreed that access was still a good idea and wanted to continue having access, respectively (Table 4). At baseline, patients planned to use and access their records over the 6-month intervention period (n=35), and did so because they were curious (n=25), wanted to see test results (n=26) and know about their health (n=18), they have a right to see what is in their record (n=16), remember what happened at a clinic visit (n=12), be sure they understood what the health professional said (n=10), and know what the professional was thinking (n=6). Over the 6-month intervention, eight people did not log into Patient Access at all. Reasons for not doing so were that they had forgotten they had access (n=3), they did not need to see test results (n=2), no particular reason (n=3), and due to worry about the privacy of information (n=1).

**Table 4:** Perceptions of and engagement with Patient Access by the intervention group at follow-up (having had access for 6 months).

			Value, n (%)
**In general, making EHRs^a^ available to people with CF^b^ (ie, having Patient Access) is a good idea? (3 people did not answer)**			
	Disagree	0 (0)	
	Somewhat disagree	0 (0)	
	Somewhat agree	2 (5)	
	Agree	39 (93)	
	Don’t know	1 (2)	
**Did you log in and access your EHR at any point during the past 6 months? (** **4 people did not answer)**			
	No	8 (20)	
	Yes	33 (80)	
**Why might you like to be able to read your EHRs (tick/cross all that apply)?**			
	I was curious	25 (56)	
	I have the right to see what’s in my medical record	16 (36)	
	I wanted to see the diagnosis/ test results	26 (58)	
	I wanted to know what my health professional was thinking	6 (13)	
	I wanted to check my record for accuracy	5 (11)	
	I wanted to be sure I understood what the health professional said	10 (22)	
	I wanted to remember what happened during the visit	12 (27)	
	I wanted to know about my health	18 (40)	
	No particular reason	0 (0)	
	Other	3 (7)	
**Why did you not access your EHRs (tick/cross all that apply)?**			
	I didn’t think it would be useful	0 (0)	
	I do not use the internet very much	0 (0)	
	I thought reading the record would make me nervous or anxious	0 (0)	
	I do not want to think about my health more than I have to	0 (0)	
	I am afraid I will find out something bad about my health that I didn’t know	0 (0)	
	I do not need to see what health care professionals wrote about my visit	0 (0)	
	I forgot I could access my health care records online	3 (7)	
	I worry about the privacy of information	1 (2)	
	I do not need to see test results	2 (4)	
	No particular reason	3 (7)	
	Other	3 (7)	
**How easy was it to understand your health care records? (** **5 people not answer)**			
	Very difficult	0 (0)	
	Somewhat difficult	3 (8)	
	Someway easy	12 (30)	
	Very easy	20 (50)	
	Don’t know	5 (12)	
**Did you ever contact the CF unit about something in your health care record? (** **6 people did not answer)**			
	Yes	5 (13)	
	No, I did not feel any need to	32 (84)	
	I considered it, but decided not to	1 (3)	
	Don’t know/ don’t remember	0 (0)	
**(If applicable) Why did you decide to contact your health care professional about something in your health care records (** **38 people did not answer)**			
	I wanted an explanation, for example, of a test result	4 (57)	
	I wanted something removed from my record	0 (0)	
	I wanted to report something I thought was an error in my record	1 (14)	
	I wanted to discuss something I disagreed with	0 (0)	
	Another reason	2 (29)	
**Were you satisfied with the health care professional’s response to your request? (** **38 people did not answer)**			
	Yes	7 (100)	
	Somewhat	0 (0)	
	No	0 (0)	
**Did you show, discuss, or share your health care record with other people? (** **6 people did not answer)**			
	Yes	27 (69)	
	No	11 (28)	
	Don’t know/don’t remember	1 (3)	
**With whom do you show/ discuss/share your health care records? (** **6 people did not answer)**			
	My partner	21 (54)	
	A family friend or relative	23 (59)	
	A friend	4 (10)	
	A doctor (outside of cystic fibrosis care)	3 (8)	
	A nurse or health care professional (outside of cystic fibrosis care)	2 (5)	
	Someone else	2 (5)	
**I would like to continue having access to my EHR (** **5 people did not answer)**			
	Yes	39 (98)	
	No	1 (2)	
**I understand my health and medical conditions better? (** **5 people did not answer)**			
	Disagree	0 (0)	
	Somewhat disagree	2 (5)	
	Somewhat agree	8 (20)	
	Agree	26 (65)	
	Don’t know	4 (10)	
**I remember to plan for my care better (** **2 people did not answer)**			
	Disagree	1 (2)	
	Somewhat disagree	3 (7)	
	Somewhat agree	11 (26)	
	Agree	21 (49)	
	Don’t know	4 (9)	
	Don’t know	3 (7)	
**I feel more in control of my health care (** **5 people did not answer)**			
	Disagree	0 (0)	
	Somewhat disagree	6 (15)	
	Somewhat agree	7 (18)	
	Agree	26 (65)	
	Don’t know	1 (2)	
**I am concerned about my privacy (** **5 people did not answer)**			
	Disagree	26 (65)	
	Somewhat disagree	11 (28)	
	Somewhat agree	2 (5)	
	Agree	0 (0)	
	Don’t know	1 (2)	
**I am concerned about the security of my record (** **5 people did not answer)**			
	Disagree	26 (65)	
	Somewhat disagree	9 (23)	
	Somewhat agree	4 (10)	
	Agree	0 (0)	
	Don’t know	1 (2)	
**I felt offended (** **5 people did not answer)**			
	Disagree	34 (85)	
	Somewhat disagree	3 (8)	
	Somewhat agree	2 (5)	
	Agree	0 (0)	
	Don’t know	1 (2)	
**Did reading your EHRs, specifically the consultations, change the way you feel about health care professionals? (** **5 people did not answer)**			
	Yes, felt much worse	0 (0)	
	Yes, felt much better	9 (23)	
	No	25 (62)	
	Don’t know	6 (15)	
**(At the start of the study) I planned to use and access to my EHR in the next 6 months (** **5 people did not answer)**			
	Strongly disagree	0 (0)	
	Disagree	0 (0)	
	Neither agree nor disagree	5 (13)	
	Agree	6 (15)	
	Strongly agree	29 (72)	

^a^EHR: electronic health care record.

^b^CF: cystic fibrosis.

Patient Access scored 86% for satisfaction, 82% for ease of use, and 80% for usefulness. The median number of logins over the study period was 9 (range 1-205). For those who did use Patient Access, patients agreed that they understood their CF better (n=34), felt more in control of their health (n=33), that information was easy to understand (n=32), and they could plan for their health condition better (n=32). There were no privacy or security concerns reported by 37 and 35 participants, respectively. Reading the consultations did not impact the way 25 participants felt about health care professionals; 9 participants felt much better, and 6 participants had not considered whether their feelings had changed. Seven patients contacted the CF Unit to discuss something in their record, with four people wanting an explanation of a test result, one patient wanted to report something they thought was an error, and two patients cited another reason not provided on the questionnaire. All were satisfied with the health care professional’s response. Two-thirds of patients (n=27) had shown, discussed, or shared their record with other people, with the majority reporting sharing it with their partner (n=21) or family member (n=23). Five participants had shown it to health care professionals outside of CF.

## Discussion

### Principal Findings

It was hypothesized that providing Patient Access would have an effect on levels of anxiety and QoL scores in the intervention group. A total of 91 adults with CF completed the 6-month study, and the median number of logins for those in the intervention group over the study period was 9 (range 1-205). There was no effect of Patient Access in the intervention group on levels of anxiety, all symptom QoL scales (respiratory, weight, and digestion), and seven QoL domains (physical, vitality, emotional, role, body image, eating disturbances, and treatment burden).

In contrary to the literature [26], levels of anxiety were not increased in our population and may reflect the chronic nature of the disease and the regular and close communication with a familiar multidisciplinary team. The slight decrease in anxiety in the intervention group after 6 months supports that Patient Access may decrease anxiety among patients [27].

At follow-up, the QoL domain “social functioning” had decreased by six points in the intervention but remained stable in the control group. To date, no other literature has reported a decrease in social functioning in response to Patient Access. This QoL domain measures how CF affects a person’s ability to participate in social activities, maintain relationships, and engage in social interactions. Viewing their health care record may have made the patient reflect on their health status and decreased their participation in activities, impacting social interactions and relationships. It is unknown if any participants were experiencing a pulmonary exacerbation, associated with a decline in lung function, hospitalizations, and reduced QoL [28], at the time of follow-up, which may have contributed to the decrease in social functioning. However, as the respiratory domain increased at follow-up for the intervention group, this reason is unlikely.

Interestingly, the QoL health perceptions domain score had decreased in the intervention by six points but increased in the control group by nine points at follow-up. The decrease in the intervention group might reflect the improved understanding of their CF and ability to review their health status. In contrast, the control group might have been optimistic about their health, potentially due to an improvement in physical functioning, and adopting a coping style that has been shown to be an independent predictor of survival in CF [29].

Although all patients had relatively high activation levels (knowledge, skills, and confidence in managing their own health and care) at baseline, and were similar between groups, the results from the PAM-13 questionnaire suggest that having access to their EHR may help sustain and reinforce this. At follow-up, those in the control group had significantly lower activation levels than the intervention group. This supports the literature showing that Patient Access can empower individuals in managing their own health [5,30,31].

Research suggests that uptake of Patient Access is affected by privacy and security concerns [6,32]. In our cohort, this was true for one participant in the intervention group who never accessed their record for this reason. Nevertheless, the opportunity to access their records was positively received by the majority of the patients who completed the study. In addition, the percentage of people who had privacy and security concerns, respectively, decreased over the 6 months (10/50, 20% of participants and 13/50, 26% of participants vs 2/40, 5% of participants and 4/40, 10% of participants).

On average, participants in the intervention group accessed their records more than once a month, which suggests that information contained in their EHR may need to be accessed outside of their clinic visit, and the convenience reduces the need for the patient to contact the CF unit. Increased workload is cited in the literature as a concern by health care professionals as a result of patients having access [3]. Seven patients contacted the CF Unit during the intervention in relation to their records. Although the method of communication (telephone, text, email, and face-to-face) or impact on the health care professional’s workload is unknown, it is positive that only a relatively small percentage of patients made contact, and all patients were satisfied with the professional’s response.

The level of trust in, and interactions with, their health care professionals and levels of self-efficacy (confidence) in self-care and self-management did not change for the majority of those with access. For those who had considered the impact of reading consultations, 22% said they felt much better toward health care professionals afterwards, therefore enhancing patient-provider communication. However, it is unknown if health care professionals altered their consultation writing style, knowing patients would now be able to view their records [33,34].

Over the next few years, there is likely to be a significant change in the sharing of medical information between patients and their health care providers. This transference of key information will be bidirectional and include clinical and physiological data collected on individual mobile devices within the home and work environment. In the United Kingdom, secure access to personal health records is now well established in primary care. However, access to secondary care records remains limited, due in part to the underinvestment in hospital IT infrastructure and the presence of multiple legacy systems. A key driving force for the expansion of digital access is the need to deliver health information transparency, improve health care, quality of records, and empower patients to comanage their own health [35-37]. Changes in digital information exchange of personal medical information have the potential to have positive effects, such as greater patient empowerment, ownership, and improved knowledge of the underlying conditions [30,32,38,39]. This was reflected in this study, with patients perceiving that digital access aided them to better understand their condition, plan for their care better, and feel more in control of their health, while providing medical information that was relatively easy to understand and proved more helpful than confusing.

The use of digital access to EHR is likely to vary between individuals, with uptake being influenced by accessibility, digital literacy, health condition, and age of the user. Previous studies have reported improved patient engagement following the introduction of digital access to EHR, although the design and function of the portal can be perceived as nonpatient-centric and inadequate by users [40]. In this study, patients gave a high score for satisfaction, with ease of use and usefulness slightly lower, suggesting that improvement in usability and functions may be needed. All but one patient in the intervention group agreed that Patient Access was a good idea and wished to continue with access after the 6 months. Since the end of the intervention, access to EHR has been granted to all adult patients with CF at the Leeds CF unit who wish to have this resource.

### Strengths and Limitations

In contrast to primary care, secondary care is in the early stages of offering full digital access. This is the first study to provide secure access to secondary care records in CF, which has incorporated the feedback about which aspects of their EHR people with CF wish to access and priorities for development [41].

Participants were mostly White British, which is reflective of CF being a Caucasian disease [42], and in young-middle adulthood. These factors may have influenced the positive uptake of the app, as older adults are less likely to use digital services [43,44]. The findings from the PHWSUQ suggest that ease of use and usefulness could be improved and highlight the importance of having an intuitive, simple interface [45]. However, as Patient Access is a UK-based mobile app, updating the app functionality is outside the remit of the research team.

### Conclusions

Our results suggest that increased patient information sharing through Patient Access to EHR is beneficial and desirable to patients, and should be implemented in other disease areas in secondary care where possible. There was no evidence of an effect of Patient Access on levels of anxiety and overall QoL, but improved subjective understanding, engagement, and control in the management of their CF. Prospective studies are needed to investigate the long-term effect of such interventions on objective health outcomes and how we can improve the functionality of such apps from the patient perspective.

## Appendix

Multimedia Appendix 1 Detailed description of questionnaires.

Multimedia Appendix 2 CONSORT eHEALTH checklist (V 1. 6. 1).

## Abbreviations

CF: cystic fibrosis
CFQ-R: Cystic Fibrosis Questionnaire-Revised
CONSORT: Consolidated Standards of Reporting Trials
EHR: electronic health care record
GAD-7: Generalized Anxiety Disorder-7
PAM-13: Patient Activation Measure-13
PEPPI: patient and provider perceived efficacy in patient-physician interaction
PHQ-9: Patient Health Questionnaire-9
PHWSUQ: Perceived Health Web Site Usability Questionnaire
QoL: quality of life

## Data Availability

The datasets analyzed during this study are available from the corresponding author on reasonable request.
