# Supplementary material for: Providing Mobile Patient Access to Their Electronic Secondary Care Patient Record in Adults With Cystic Fibrosis: Results of a Prospective, Parallel, Randomized Open-Pilot Quantitative Study
Source: JMIR Form Res. 2025 Dec 25;9:e69747. doi: 10.2196/69747 (PMC12784140; doi:10.2196/69747)
Supplement: Multimedia Appendix 1 [file formative_v9i1e69747_app1.docx]

**Providing mobile Patient Access to their electronic secondary care patient record in adults with cystic fibrosis: Results of a prospective, parallel, randomised open pilot study**

*Helen K Chadwick_a,b_, Akhil Sawant_b_, Helen White_c_, Lindsey Gillgrass_b_, Giulia Spoletini_b_, Ian J Clifton_b_, Christine Etherington_b_, Daniel G Peckham_a,b_*

1. Respiratory medicine, Leeds Institute of Medical Research at St James's, University of Leeds, Leeds, LS2 9JT, UK
2. Adult Cystic Fibrosis Unit, St James's University Hospital, Leeds Teaching Hospitals NHS Trust, Leeds, LS9 7TF, UK
3. School of Clinical & Applied Science, Leeds Beckett University, Leeds, LS1 3HE, UK

Corresponding author: Helen K Chadwick, Leeds Institute of Medical Research at St James’s, University of Leeds, Leeds, LS2 9JT, UK

Email address: H.K.Chadwick@leeds.ac.uk

**Methods - questionnaires**

Levels of anxiety; Generalised Anxiety Disorder-7 (GAD-7)

Levels of anxiety were measured using the Generalised Anxiety Disorder-7 questionnaire (GAD-7). This questionnaire has good reliability, as well as criterion, construct, factorial, and procedural validity, and is validated for use in people with CF. It consists of seven items (and one non-scored item which assigns weight to the degree to which anxiety problems have affected the patient’s level of function), assessing over the last two weeks, how often patients have been bothered by anxiety related problems. Possible scores range from 0 (not at all) to 3 (nearly every day), with total scores of 0-5, 6-10. 11-15, and 15-21 representing mild, moderate, moderately severe, and severe anxiety respectively.

Quality of life; Cystic Fibrosis Questionnaire-R (CFQ-R)

Quality of life was assessed using the Cystic Fibrosis Questionnaire- Revised (CFQ-R). The CFQ-R is a disease-specific health-related qualify of life (HRQoL) measure for people with CF and has undergone extensive reliability and validity testing. This questionnaire consists of 50 items, and assesses the following domains: Physical Functioning, Vitality, Health Perceptions, Respiratory Symptoms, Treatment Burden, Role Functioning, Emotional Functioning, and Social Functioning. Scores ranged from 0 to 100, with higher scores indicating better HRQoL.

Levels of depression; Patient Health Questionnaire-9 (PHQ-9)

Severity of depression was measured using the Patient Health Questionnaire-9 (PHQ-9) which has shown to be reliable and is validated for use in people with CF. The questionnaire is a brief (nine scored items and a non-scored item which assigns weight to the degree to which depressive problems have affected the patient’s level of function) and multipurpose instrument for screening, diagnosing, measuring, and monitoring the severity of depression in the past two weeks. Possible scores range from 0 (not at all) to 3 (nearly every day), with total scores of 0-5, 6-10, 11-15, and 15-21 representing mild, moderate, moderately severe, and severe depression respectively.

Motivation towards engaging in healthcare; Patient Activation Measure-13 (PAM-13)

The Patient Activation Measure (PAM-13) assessed the knowledge, skills and confidence a person with CF has in managing their healthcare. The questionnaire is a validated, commercially licenced tool (a US company, Insignia Health LLC) and consists of 13 items. Scores are between 0 and 100, which places the participant at one of four levels of activation, each of which reveals insight into a range of health-related characteristics, including behaviours and outcomes. The four levels of activation are:

- (0-25): Individuals tend to be passive and feel overwhelmed by managing their own health. They may not understand their role in the care process.
- (26-50): Individuals may lack the knowledge and confidence to manage their health.
- (51-75): Individuals appear to be taking action but may still lack the confidence and skill to support their behaviours.
- (76-100): Individuals have adopted many of the behaviours needed to support their health but may not be able to maintain them in the face of life stressors.

Patient and Provider Perceived Efficacy in Patient-Physician Interactions (PEPPI)

The impact on patient and provider relationships was assessed using the Perceived Efficacy in Patient-Physician Interactions questionnaire (PEPPI). The 10-item questionnaire is based on the previous work of Bandura regarding the assessment of self-efficacy, a motivator for health behaviour, and has been shown to be reliable (α =.91) and valid (discriminant and convergent). It assesses the participants’ level of trust in and interactions with their healthcare professionals (5 items on a scale of 0-5; not at all confident to very confident) and levels of self-efficacy (confidence) in self-care and self-management (5 items on a scale of 0-10; not at all confident to very confident). Scores range from 10-50, with 50 representing higher patient perceived self-efficacy.

Perceptions of and intention to engage with Patient Access; baseline questionnaire

The baseline questionnaire was split into three sections; Section I contained 7 items about what patients with CF think about having access to their EHR and reasons for accessing, and not accessing, their record. Items were modified from the OpenNotes pre survey regarding perceptions of and benefits to engage with Patient Access and issues with privacy and security, and the Physician and Patient Attitudes toward Technology in Medicine survey regarding how information is shared and who has ownership.

Section II contained eight questions about how having access to their EHR might affect them, with Likert responses on a scale from Disagree to Agree, and also includes the option of ‘Don’t know’. Items relating to security and privacy were taken from the OpenNotes Study questionnaire. Section III contained 3 items which were based on the drivers and barriers to patients’ acceptance of Patient Access based on an extension to the ‘Unified Theory of Acceptance and Use of Technology’ (UTAUT), with Likert responses of 1 to 5, where 1 = strongly disagree and 5 = strongly agree. Items were modified from a reliable and valid survey which assessed Electronic Health Record Patient Portal Adoption using UTAUT.

Perceptions of and engagement with Patient Access; end of intervention [intervention group only]

The ‘end of intervention’ questionnaire contained modified items from the baseline questionnaire to reflect having had access to their EHR for the 6 months of the study. Section I contained 11 items and were in relation to whether patients with CF have accessed their EHR, why they did/didn’t access their record, and whether they would like to continue to have access. Section II contained 6 items were about how having access to their EHR has affected them. Section III contained 2 items were about the drivers and barriers to patients’ acceptance of Patient Access.

Computer literacy

Computer literacy was assessed using items taken from the ‘My Diabetes, My way’ survey to measure the types, frequency, and experience of computer/internet use. The original ‘My Diabetes, My way’ survey was designed for potential users of the records access system and was based around the objectives of the NHS Scotland self-management strategy. Face validity of the questionnaire was assessed by independent reviewers to determine its ability to measure patient expectations of records access. Items were also selected from the modified items from the 2009 US National Health Interview Survey (NHIS) conducted by the National Center for Health Statistics (NCHS); http://www.cdc.gov/nchs/nhis.htm) included in a pre- study survey which was investigating inpatient engagement using technology.

Perceived Health Web Site Usability Questionnaire (PHWSUQ); end of intervention [intervention group only]

The Perceived Health Web Site Usability Questionnaire (PHWSUQ) has 12 items on a 7-point Likert scale from 1 (very unsatisfied/strongly disagree) to 7 (Very satisfied/strongly agree), and assesses three functionality and usability dimensions: satisfaction, ease of use, and usefulness. The measure has high internal consistency and validity. A modified version was used which excluded 2 items relating to satisfaction as these are not relevant to Patient Access: ease of listening to audio-information, and quality of video information.
