# Supplementary material for: Providing Mobile Patient Access to Their Electronic Secondary Care Patient Record in Adults With Cystic Fibrosis: Results of a Prospective, Parallel, Randomized Open-Pilot Quantitative Study
Source: JMIR Form Res. 2025 Dec 25;9:e69747. doi: 10.2196/69747 (PMC12784140; doi:10.2196/69747)
Supplement: Multimedia Appendix 2 [file formative_v9i1e69747_app2.docx]

| PROVIDING MOBILE PATIENT ACCESS TO THEIR ELECTRONIC SECONDARY CARE PATIENT RECORD IN ADULTS WITH CYSTIC FIBROSIS: RESULTS OF A PROSPECTIVE, PARALLEL, RANDOMISED OPEN PILOT STUDY | |
| --- | --- |
| Manuscript Number 69747 | |
| **Question** | **Response** |
| **TITLE**  **Identification as a randomized trial in the title** | |
| TITLE  1a-i) Identify the mode of delivery in the title | The title refers to the app 'Patient Access', in which patients can see their electronic patient record –  "PROVIDING MOBILE PATIENT ACCESS TO THEIR ELECTRONIC SECONDARY CARE PATIENT RECORD” |
| 1a-ii) Non-web-based components or important co-interventions in title | Patient Access is the intervention, and included in the title. How we tested the effectiveness of having access is not included in the title e.g., questionnaires. But we do state that the manuscript reports the results of the intervention. |
| 1a-iii) Primary condition or target group in the title | We clearly state that the target group are “ADULTS WITH CYSTIC FIBROSIS” (CF) |
| **ABSTRACT**  **Structured summary of trial design, methods, results, and conclusions** | |
| 1b-i) Key features/ functionalities /components of the intervention and comparator in the METHODS section of the ABSTRACT | We clearly describe that the intervention compares patients with cystic fibrosis receiving access to their electronic healthcare record or having no access.  We do not provide all the components of Patient Access as it is widely available in primary care. |
| 1b-ii) Level of human involvement in the METHODS section of the ABSTRACT. | We provide background that people with CF were recruited on a consecutive basis, from outpatient clinics or as an inpatient on the regional Leeds adult CF unit.  We state " At baseline and six months, paper-based self-report questionnaires were completed by participants” …  “Once baseline questionnaires were completed, participants were randomised into either the intervention or control group (1:1), with those in the intervention given instructions about how to gain access and the functions of Patient Access by the research team” |
| 1b-iii) Open vs. closed, web-based (self-assessment) vs. face-to-face assessments in the METHODS section of the ABSTRACT | We report that “People with CF were recruited on a consecutive basis, from outpatient clinics or as an inpatient on the regional Leeds adult CF unit.”  We used questionnaires, at two time points, to evaluate the effect of having access to their secondary care medical records- " At baseline and six months, paper-based self-report questionnaires were completed by participants to assess having access to EHR on psychological impact, patient satisfaction, quality of life (QoL), patient and physician relationships, and pattern and rates of adherence to treatment. Perceptions and engagement with Patient Access, and computer literacy were also assessed". |
| 1b-iv) RESULTS section in abstract must contain use data | We report the number of patients with cystic fibrosis who completed the pilot study in total, and in both groups (intervention and control) and provide data about median age and number of males; “Ninety-one people with CF completed the six-month study. Intervention n=45, median age=27.5 (IQR 12.0) years, 22 males. Control group n=46, median age=27.0 (IQR 15.0) years, 29 males. Median number of logins was nine (range 1-205).”  We report the median number of logins to Patient Access “Median number of logins was nine (range 1-205)”.  We also report the percentage of those who wish to continue having access to their secondary care records via Patient Access. “Of those who had EHR access, 41/42 participants (98%) agreed that access to EHR should continue.” |
| 1b-v) CONCLUSIONS /DISCUSSION in abstract for negative trials | Although our intervention was negative (it did not increase levels of anxiety or decrease QoL), this is a positive finding as the literature suggests that levels of anxiety may increase due to having access to their medical records.  We state in the abstract " This pilot study suggests that providing access to EHR in adults with CF does not appear to have a negative effect (increase levels of anxiety or decrease QoL) and uptake by patients has been very positive.” |
| **INTRODUCTION**  **Background and objectives** | |
| 2a-i) Problem and the type of system/solution  *Scientific background and explanation of rationale* | We provide background that Patient Access in secondary care is limited due to the "accessibility of platforms and the fragmentation and lack of digitised records". And that the rollout in Primary Care has met some resistance from healthcare professionals because of "increased workload, patient anxiety and potential litigation".  We state that, because the "regional adult and paediatric cystic fibrosis (CF) services introduced a modified primary care electronic healthcare record (EHR) in 2007", we have the ability to provide access in secondary care. However, like primary care, the multidisciplinary team had concerns about increases in levels of anxiety and the way the team recorded information, and therefore conducted a pilot study.  Our aims were to "evaluate the feasibility, benefits, and acceptability to patients of providing secure access of linked secondary care in CF...explore technological usability and the impact of the shared records on communication and patient satisfaction.” |
| 2a-ii) Scientific background, rationale: What is known about the (type of) system | Patients with cystic fibrosis registered to the Leeds services have a “modified primary care electronic healthcare record (EHR)” and have the ability to have ‘Patient Access’.  Due to concerns by the multidisciplinary team, we provided access to a sample of patients to evaluate the effects, before rolling it out to all patients. |
| 2b) Specific objectives or hypotheses | We clearly state our objectives and hypothesis:  "prior to opening the portal to all patients, we performed a pilot study to evaluate the feasibility, benefits, and acceptability to patients of providing secure access of linked secondary care in CF. We also wanted to explore technological usability and the impact of the shared records on communication and patient satisfaction."  “It was hypothesised that providing Patient Access would have an effect on levels of anxiety and quality of life scores in the intervention group.” |
| **METHODS**  **Trial design** | |
| 3a) CONSORT: Description of trial design (such as parallel, factorial) including allocation ratio section | We clearly define the pilot study in the Design " A prospective, parallel, randomised, open, pilot study " |
| 3b) CONSORT: Important changes to methods after trial commencement (such as eligibility criteria), with reasons | n/a - We did not implement any changes. |
| 3b-i) Bug fixes, Downtimes, Content Changes  *Important changes to methods after trial commencement (such as eligibility criteria), with reasons* | As the pilot utilised an app which is already available on iOS and Android, there were no content changes or downtimes implemented by the research team. |
| **Participants** | |
| 4a) CONSORT: Eligibility criteria for participants | We clearly define eligibility in the Design section  "People with CF were recruited on a consecutive basis, either from outpatient clinics or as an inpatient on the Regional Leeds Adult CF Unit. Eligible people were identified using the Leeds Adult CF Unit electronic healthcare records (EHR; 8) Inclusion criteria consisted of diagnosis of CF (confirmed by the presence a CF phenotype with either two CF causing mutations or a single mutation with two positive sweat chloride (>60 mmol/L)), age 17 years or over, able to give written informed consent, and presence of an electronic healthcare record (EHR) system at the Regional Leeds Adult CF Unit. Patients were excluded if they were taking part in a clinical trial." |
| 4a-i) Computer / Internet literacy | We measured computer literacy in our pilot using a questionnaire modified from the “My diabetes, my way” survey to assess types, frequency, and experience of computer/internet use” |
| 4a-ii) Open vs. closed, web-based vs. face-to-face assessments:  *Mention how participants were recruited (online vs. offline), e.g., from an open access website or from a clinic, and clarify if this was a purely web-based trial, or there were face-to-face components (as part of the intervention or for assessment), i.e., to what degree the study team got to know the participant.* | We report that patients were recruited "on a consecutive basis, either from outpatient clinics or as an inpatient on the Regional Leeds Adult CF Unit”, therefore face-to-face.  We provide a description of the questionnaires in the Measures section of the methods including the format: “paper-based self-report questionnaires”  Given the service, the research team/ authors know the patients very well (outpatient appointments every 6-8 weeks, since the age of 16/17, and during inpatient stays when treatment is required). We report at the start of the Results section how many participants met the eligibility criteria and were recruited in each group (intervention and control) |
| 4a-iii) Information giving during recruitment We state in the Procedures section  *Specify how participants were briefed for recruitment and in the informed consent procedures, as this information may have an effect on user self-selection, user expectation and may also bias results.* | "Eligible adults with CF were approached face-to-face at either routine cystic fibrosis outpatient clinics (Seacroft Hospital, Leeds, UK) or as an inpatient on the regional Leeds adult CF unit (St James’s University Hospital, Leeds, UK). If they expressed interest, they were provided with a participant information sheet by a member of the research team (who was also part of their direct care team), the study was explained to them, and they had the opportunity to ask questions and have them answered satisfactorily. Upon enrolment, participants completed the paper consent form and baseline paper-based self- report questionnaires.”  After randomisation, “Those in the intervention group were instructed in how to gain access and the functions of Patient Access explained to them by a member of the research team (who was also part of their direct care team); access to current problems and medication, test requests, letter, consultations, allergies and immunisations. Information about Patient Access security and privacy was also explained. No prompts were used in the study; those in the intervention used Patient Access at their discretion during the 6-month study period. Help and support was provided only if the participant requested this. After 26 (+/-1) weeks, participants completed the follow-up paper-based questionnaires. Those in the intervention group also completed the end of intervention ‘Perception of Patient Access and their engagement’ questionnaire and PHWSUQ on paper. Following successful completion of the questionnaires, participants in the control group were granted access to their EHR if they expressed interest." |
| 4b) CONSORT: Settings and locations where the data were collected | We state, in the Design subsection, that " People with CF were recruited on a consecutive basis, either from routine cystic fibrosis outpatient clinics (Seacroft Hospital, Leeds, UK) or as an inpatient on the regional Leeds adult CF unit (St James’s University Hospital, Leeds, UK)” |
| 4b-i) Report if outcomes were (self-)assessed through online questionnaires | We report that outcome measures were assessed using “paper-based self-report questionnaires”. |
| 4b-ii) Report how institutional affiliations are displayed | Study documentation contained the logos of institutions relevant to the study.  As the pilot used an already published app (Patient Access), no affiliations were displayed in relation to this specific study. |
| **Interventions**  **5) CONSORT: Describe the interventions for each group with sufficient details to allow replication, including how and when they were actually administered** | |
| 5-i) Mention names, credential, affiliations of the developers, sponsors, and owners | None of the authors had any conflicts of interest. |
| 5-ii) Describe the history/development process  *of the application and previous formative evaluations (e.g., focus groups, usability testing), as these will have an impact on adoption/use rates and help with interpreting results.* | Stated in the Patient and public involvement in research section at the end of the manuscript, " Previous research [by the authors] sought patient feedback regarding which aspects of their EHR people with CF wish to access, and their priorities for development. This was incorporated into the design of this study. People with CF were also involved in the design of this study and were asked to assess the burden of questionnaire completion.” |
| 5-iii) Revisions and updating | Patient Access is not owned or developed by the authors. It is provided by EGTON MEDICAL INFORMATION SYSTEM LIMITED. |
| 5-iv) Quality assurance methods | This item is not relevant to the study |
| 5-v) Ensure replicability by publishing the source code, and/or providing screenshots/screen-capture video, and/or providing flowcharts of the algorithms used | Patients were able to access their cystic fibrosis electronic healthcare record using the Patient Access app, which was already developed and widely used in the UK. |
| 5-vi) Digital preservation | The authors utilised an already created resource, Patient Access, created by EGTON MEDICAL INFORMATION SYSTEM LIMITED. Examples pages are displayed on the app store pages (iOS and Android). |
| 5-vii) Access | Participants had to be registered to the Leeds cystic fibrosis services to participate. The app was free, but required a valid electronic medical record as stated in the eligibility criteria in the Design section; "and presence of an electronic healthcare record (EHR) system at the Regional Leeds Adult CF Unit". Participants accessed the app as and when needed. We evaluated for what reasons in a follow-up questionnaire at the end of the intervention for those who had Patient Access. "Those in the intervention group were instructed in how to gain access and the functions of Patient Access explained to them." |
| 5-viii) Mode of delivery, features /functionalities/ components of the intervention and comparator, and the theoretical framework | The pilot utilised an app developed by EMIS, called Patient Access. Participants were only allowed to view their own cystic fibrosis electronic healthcare record. |
| 5-ix) Describe use parameters | People with cystic fibrosis were advised to use the app as and when they needed, after the different functionalities were explained. |
| 5-x) Clarify the level of human involvement As stated in the subsection, procedures, | "Those in the intervention group were instructed in how to gain access and the functions of Patient Access explained to them by a member of the research team (who was also part of their direct care team); access to current problems and medication, test requests, letter, consultations, allergies and immunisations. Information about Patient Access security and privacy was also explained. No prompts were used in the study; those in the intervention used Patient Access at their discretion during the 6-month study period. Help and support was provided only if the participant requested this."  The same process would be followed outside the intervention/ study. |
| 5-xi) Report any prompts/reminders used No prompts were used in the study. | People with cystic fibrosis (in the intervention) used Patient Access as and when required for the 6 month period – “No prompts were used in the study; those in the intervention used Patient Access at their discretion during the 6-month study period. Help and support was provided only if the participant requested this." |
| 5-xii) Describe any co-interventions (incl. training/support) | As stated above, and in the subsection ‘procedures’:  "Those in the intervention group were instructed in how to gain access and the functions of Patient Access explained to them by a member of the research team (who was also part of their direct care team); access to current problems and medication, test requests, letter, consultations, allergies and immunisations. Information about Patient Access security and privacy was also explained. No prompts were used in the study; those in the intervention used Patient Access at their discretion during the 6-month study period. Help and support was provided only if the participant requested this".  The same process would be followed outside the intervention/ study. No other training and/or support was provided unless a participant requested this to a member of the multidisciplinary team. |
| **Outcomes** | |
| 6a) CONSORT: Completely defined pre-specified primary and secondary outcome measures, including how and when they were assessed | The manuscript clearly defines the primary and secondary outcome measures, and the supplementary material contains a more detailed description. It also specifies that questionnaires were completed at week 0, and again at week 26±1. |
| 6a-i) Online questionnaires: describe if they were validated for online use and apply CHERRIES items to describe how the questionnaires were designed/deployed The study did not use online questionnaires. | Not applicable |
| 6a-ii) Describe whether and how “use” (including intensity of use/ dosage) was defined /measured/ monitored | Objective data were collected about the number of logins over the 6 month period.  We also collected subjective data about the reasons why people used Patient Access e.g., to see test results. We also report the reasons why participants did not use Patient Access - "they had forgotten they had access (n=3), they did not need to see test results (n=2), no particular reason (n=3), and due to worry about the privacy of information (n=1)". |
| 6a-iii) Describe whether, how, and when qualitative feedback from participants was obtained | Qualitative feedback was not sought as part of the pilot. We had already gathered information about what aspects of Patient Access people would like to access to in previous research; this is stated in the patient and public involvement in research section.  We did gain quantitative feedback about the 'Perceptions of and intention to engage with Patient Access' at baseline (for both intervention and control group) and follow-up (intervention) though. Some of the questions did include an 'other' response, where participants could specify their reason/answer. |
| 6b) CONSORT: Any changes to trial outcomes after the trial commenced, with reasons | There were no changes to the trial outcomes after the pilot study had commenced. |
| **7a) CONSORT: How sample size was determined** | |
| 7a-i) Describe whether and how expected attrition was taken into account when calculating the sample size | " An a priori sample size calculation was performed using the following formula, assuming a 2-sided 5% significance level, a standardised effect size of 0.6, 80% power, and a drop-out rate of 10%. The calculation indicated that 100 participants would be required, allocated to group on a 1 intervention: 1 control, resulting 50 individuals being recruited to the intervention (given mobile access to their own secondary care EHR) and 50 people to the control group (no access to their secondary care EHR)".  "Three hundred and fifty people met the inclusion criteria. In total, 102 patients were recruited, 51 in each group (see Figure 1). An extra individual was recruited to each group to account for two people who dropped out shortly after consent and randomisation, respectively" |
| 7b) CONSORT: When applicable, explanation of any interim analyses and stopping guidelines | No interim analyses or stopping guidelines were used in the study; this was a pilot with a commercially available app. |
| **Sequence generation** | |
| 8a) CONSORT: Method used to generate the random allocation sequence | "Random sequenced numbers were generated with the smallest value of 1 and largest of 100, and even numbers assigned to the intervention." |
| 8b) CONSORT: Type of randomisation; details of any restriction (such as blocking and block size) | "Once consented, individuals were allocated to either the intervention or control group (1:1 ratio) by the research team using a randomised sampling technique."  "The [sample size] calculation indicated that 100 participants would be required, allocated to group on a 1 intervention: 1 control, resulting 50 individuals being recruited to the intervention (given online access to their own secondary care EHR) and 50 people to the control group (no access to their secondary care EHR)." |
| **Allocation concealment mechanism** | |
| 9) CONSORT: Mechanism used to implement the random allocation sequence (such as sequentially numbered containers), describing any steps taken to conceal the sequence until interventions were assigned | "Random sequenced numbers were generated (https://www.random.org/sequences/) with the smallest value of 1 and largest of 100, and even numbers assigned to the intervention." A spreadsheet dictated which arm the next recruited participant was assigned to. |
| **Implementation** | |
| 10) CONSORT: Who generated the random allocation sequence, who enrolled participants, and who assigned participants to interventions | This was done by the authors/ research team. |
| **11a) CONSORT: Blinding - If done, who was blinded after assignment to interventions (for example, participants, care providers, those assessing outcomes) and how** | |
| 11a-i) Specify who was blinded, and who wasn’t | The authors and participants were not blinded. |
| 11a-ii) Discuss e.g., whether participants knew which intervention was the “intervention of interest” and which one was the “comparator” | "Upon enrolment, participants completed the paper consent form and baseline paper-based self- report questionnaires in the following order: GAD-7, PHQ-9, PAM-13, CFQ-R, PEPPI, computer literacy and ‘perceptions of and intention to engage with Patient Access’. Participants were then randomised into either the intervention or control group."  Given the nature of the pilot study, it was obvious to the participants if they were assigned the intervention or control group i.e., if they were receiving Patient Access or not. |
| 11b) CONSORT: If relevant, description of the similarity of interventions | This item is not relevant to this study. |
| 11b) CONSORT: If relevant, description of the similarity of interventions | This item is not relevant to this study. |
| **Statistical methods** | |
| 12a) CONSORT: Statistical methods used to compare groups for primary and secondary outcomes | " Analysis was conducted separately for participants with data at both baseline and follow-up. Descriptive statistics were produced for computer literacy. Data are expressed as number or median [interquartile range; IQR] as appropriate. For proportions, a Fisher’s exact test was used, and for measures a Mann-Whitney U test was used for between subjects. No correction for multiplicity was applied. P <0.05 was used as a guide to significance and all computations used IBM SPSS v26.0 or higher (IMP, Armonk, New York)." |
| 12a-i) Imputation techniques to deal with attrition / missing values | "In total, 102 patients were recruited, 51 in each group (see Figure 1). An extra individual was recruited to each group to account for two people who dropped out shortly after consent and randomisation, respectively."  Those who did not complete the trial were not included in the analyses. |
| 12b) CONSORT: Methods for additional analyses, such as subgroup analyses and adjusted analyses | This item is not relevant for this study. |
| **RESULTS**  **Participant flow (a diagram is strongly recommended)** | |
| 13a) CONSORT: For each group, the numbers of participants who were randomly assigned, received intended treatment, and were analysed for the primary outcome | "In total, 102 patients were recruited, 51 in each group (see Figure 1). An extra individual was recruited to each group to account for two people who dropped out shortly after consent and randomisation, respectively. ... Groups were similar for age (intervention: 27.5 (IQR 14.0) years vs. control 27 (IQR 15.0) years, P =.96) and sex distribution (intervention 27 males, control 32 males, P =.32).” |
| 13b) CONSORT: For each group, losses and exclusions after randomisation, together with reasons | A CONSORT flow diagram (figure 1) is included within the manuscript.  We report the number who completed the intervention in the 'follow-up' section of the results. |
| 13b-i) For each group, losses and exclusions after randomisation, together with reasons; Attrition diagram | We do not include any log in data once the pilot study ended. |
| **Recruitment** | |
| 14a) CONSORT: Dates defining the periods of recruitment and follow-up | " A prospective, parallel, randomised, open, pilot study was conducted between April 2018 and May 2019 ". |
| 14a-i) Indicate if critical “secular events” fell into the study period | No secular events occurred during the study period. |
| 14b) CONSORT: Why the trial ended or was stopped (early) | This item is not relevant; the trial was not stopped early. The trial was a 6 month pilot study. |
| **Baseline data** | |
| 15) CONSORT: A table showing baseline demographic and clinical characteristics for each group | These data are displayed in Table 1 and 2. |
| 15-i) Report demographics associated with digital divide issues | Table 2 reports the data for computer literacy. Groups were similar for this outcome measure. |
| **Numbers analysed**  16a) CONSORT: For each group, number of participants (denominator) included in each analysis and whether the analysis was by original assigned groups | |
| 16-i) Report multiple “denominators” and provide definitions | We report the number of participants at baseline, and again at follow up in the Results. In the tables, we report the number of patients who provided missing data for specific questions e.g. Tables 2 and 4. |
| 16-ii) Primary analysis should be intent-to-treat | We have not performed intention-to-treat analysis. |
| **Outcomes and estimation** | |
| 17a) CONSORT: For each primary and secondary outcome, results for each group, and the estimated effect size and its precision (such as 95% confidence interval) | We report these data in table 1. |
| 17a-i) Presentation of process outcomes such as metrics of use and intensity of use | "... for the intervention group only, data relating to frequency of logins to their records were collected." "The median number of logins over the study period was nine (range 1 to 205)." |
| 17b) CONSORT: For binary outcomes, presentation of both absolute and relative effect sizes is recommended | We have not presented effect sizes for results. |
| **Ancillary analyses**  18) CONSORT: Results of any other analyses performed, including subgroup analyses and adjusted analyses, distinguishing pre-specified from exploratory | |
| 18-i) Subgroup analysis of comparing only users | We have not performed any subgroup analyses. |
| 18-i) Subgroup analysis of comparing only users | We have not performed any subgroup analyses. |
| **Harms**  19) CONSORT: All important harms or unintended effects in each group | |
| 19-i) Include privacy breaches, technical problems | We report that Patient Access did not have an effect on " levels of anxiety, all symptom QoL scales (respiratory, weight, digestion) and seven QoL domains (physical, vitality, emotional, role, body image, eating disturbances, treatment burden)”; The primary outcome measures.  This is in contrast to literature which suggests e.g., levels of anxiety increase, and levels of trust in healthcare professionals may decrease.  There were no privacy breaches or known technical problems during the study. In terms of privacy, one person in the intervention group did not use Patient Access due to this reason. However, “the percentage of people who had privacy and security concerns respectively decreased over the six months (20% and 26% vs 4% and 9%).  "Patients gave a high score for satisfaction, with ease of use and usefulness slightly lower, suggesting that improvement in usability and functions may be needed." However, given the app was not developed for the study, and was already available, this may reflect national user feelings. |
| 19-ii) Include qualitative feedback from participants or observations from staff/researchers | We only gained quantitative feedback from participants (patients with cystic fibrosis) about the app.  We report " In the intervention group, 41 of the 42 participants (98%) agreed that access was still a good idea and want to continue having access respectively (Table 4).  At baseline, patients planned to use and access their records over the 6-month intervention period (n=35), and did so because they were curious (n=25), wanted to see test results (n=26) and know about their health (n=18), they have a right to see what’s in their record (n=16), remember what happened at a clinic visit (n=12), be sure they understood what the health professional said (n=10), and know what the professional was thinking (n=6).  Over the six-month intervention, eight people did not log into Patient Access at all. Reasons for not doing so were because they had forgotten they had access (n=3), they did not need to see test results (n=2), no particular reason (n=3), and due to worry about the privacy of information (n=1).” |
| **DISCUSSION**  **20) CONSORT: Trial limitations, addressing sources of potential bias, imprecision, multiplicity of analyses** | |
| 20-i) Trial limitations, addressing sources of potential bias, imprecision | Participants in the study were not blinded. We report on use of Patient Access across the 6 month period including reasons for non-use. We also acknowledge that the participants have close communication with the familiar multidisciplinary team, which might have affected levels of anxiety. |
| **21) CONSORT: Generalisability (external validity, applicability) of the trial findings** | |
| 21-i) Generalizability to other populations | “Our results suggest that increased patient information sharing through Patient Access to [electronic healthcare records is beneficial and desirable to patients, and should be implemented in other disease areas in secondary care where possible.”  We acknowledge that the participants are a specific population with a chronic disease who have close communication with the familiar multidisciplinary team, which might have affected the results.  “Participants were mostly White British, which is reflective of CF being a Caucasian disease [43], and in young-middle adulthood. These factors may have influenced the positive uptake of the app, as older adults are less likely to utilise online services.”  However, “this is the first study to provide secure access of secondary care records in CF which has incorporated the feedback about which aspects of their EHR people with CF wish to access and priorities for development”.  Since the end of the intervention, access to Patient Access has been granted to all adult patients with cystic fibrosis at the Leeds unit who wish to have this resource. The intervention utilised the Patient Access app, which is free and available for download on iOS and Android. " In the present study, patients gave a high score for satisfaction, with ease of use and usefulness slightly lower, suggesting that improvement in usability and functions may be needed”. Any developments would have implications to all users. |
| 21-ii) Discuss if there were elements in the RCT that would be different in a routine application setting | "Following successful completion of the [follow-up ] questionnaires, participants in the control group were granted access to their [electronic healthcare record] if they expressed interest." These participants were given the same training as the intervention group.  "Since the end of the intervention, access to Patient Access has been granted to all adult patients with CF (cystic fibrosis) at the Leeds CF unit who wish to have this resource." |
| **22) CONSORT: Interpretation consistent with results, balancing benefits and harms, and considering other relevant evidence** | |
| 22-i) Restate study questions and summarize the answers suggested by the data, starting with primary outcomes and process outcomes (use) | We restate the study hypothesis, summarise the number of participants who took part, and the results at the start of the discussion.  “It was hypothesised that providing Patient Access would have an effect on levels of anxiety and quality of life scores in the intervention group. Ninety-one adults with CF completed the six-month study, and the median number of logins for those in the intervention group over the study period was nine (range 1 to 205). There was no effect of Patient Access in the intervention group on levels of anxiety, all symptom QoL scales (respiratory, weight, digestion) and seven QoL domains (physical, vitality, emotional, role, body image, eating disturbances, treatment burden).” |
| 22-ii) Highlight unanswered new questions, suggest future research | We discuss the implications for clinical practice, and suggest that future research is needed - "Prospective studies are needed to investigate the long-term effect of such interventions on objective health outcomes and how we can improve the functionality of such application from the patient perspective."  We also suggest that Patient Access should be implemented in other disease areas where possible “Our results suggest that increased patient information sharing through Patient Access to EHR is beneficial and desirable to patients, and should be implemented in other disease areas in secondary care where possible.” |
| **OTHER INFORMATION**  **Registration**  **Protocol**  **Funding**  **Competing interests** | |
| 23) CONSORT: Registration number and name of trial registry | ClinicalTrials.gov Identifier: NCT06122025 |
| 24) CONSORT: Where the full trial protocol can be accessed, if available | The information included within the protocol has been uploaded to the ClinicalTrials.gov Identifier: NCT06122025 |
| 25) CONSORT: Sources of funding and other support (such as supply of drugs), role of funders | "This research was supported by a UK Cystic Fibrosis Trust Clinical Excellence and Innovation Award. Award/Grant number is not applicable" |
| X26-i) Comment on ethics committee approval | In the 'design', we state "The study was approved by London - Camberwell St Giles Research Ethics Service (17/LO/19; 26/11/2017)." |
| X26-ii) Outline informed consent procedures | " Eligible adults with CF were approached face-to-face at either routine cystic fibrosis outpatient clinics (Seacroft Hospital, Leeds, UK) or as an inpatient on the regional Leeds adult CF unit (St James’s University Hospital, Leeds, UK). If they expressed interest, they were provided with a participant information sheet by a member of the research team (who was also part of their direct care team), the study was explained to them, and they had the opportunity to ask questions and have them answered satisfactorily. Upon enrolment, participants completed the paper consent form" |
| X26-iii) Safety and security procedures | "Those in the intervention group were instructed in how to gain access and the functions of Patient Access explained to them." This included security about the app etc.  The intervention also assessed privacy concerns "Research suggests that uptake of Patient Access is affected by privacy and security concerns. In our cohort this was true for one participant in the intervention group who never accessed their record for this reason. Nevertheless, the opportunity to access their records was positively received by the vast majority of the patients who completed the study. In addition, the percentage of people who had privacy and security concerns respectively decreased over the six months (20% and 26% vs. 4% and 9%)."  There is a Patient Access support page https://support.patientaccess.com/ |
| X27-i) State the relation of the study team towards the system being evaluated | The authors declare no competing interests. |
